# Correlates of supportive care needs among Asian Americans with colorectal, liver, or lung cancer from a web‐based patient navigation portal intervention: The Patient COUNTS study

**DOI:** 10.1002/cnr2.1971

**Published:** 2024-02-13

**Authors:** Katarina Wang, Janet N. Chu, Debora L. Oh, Salma Shariff‐Marco, Laura Allen, Mei‐Chin Kuo, Ching Wong, Hoan Bui, Junlin Chen, Feng Ming Li, Carmen Ma, Angeline Truong, Scarlett L. Gomez, Tung T. Nguyen, Janice Y. Tsoh

**Affiliations:** ^1^ Asian American Research Center on Health, University of California San Francisco; ^2^ Department of Epidemiology & Biostatistics University of California San Francisco; ^3^ Division of General Internal Medicine University of California San Francisco; ^4^ Helen Diller Family Comprehensive Cancer Center, University of California San Francisco; ^5^ Department of Psychiatry and Behavioral Sciences University of California San Francisco

**Keywords:** Asian American, cancer, cultural competence, cultural humility, multilingual, patient navigation, supportive care needs

## Abstract

**Background:**

Cancer is the leading cause of death among Asian Americans, who often face barriers to cancer care. Cancer supportive care needs among Asian Americans remain understudied.

**Aims:**

We examined cancer supportive care needs and participant factors correlated with these needs, identified profiles of supportive care needs, and examined whether needs profiles are associated with quality of life among Asian American adults.

**Methods and Results:**

We recruited 47 Asian American adults with colorectal, liver, or lung cancer who spoke Chinese, English, or Vietnamese, and were starting or undergoing cancer treatment. We assessed cancer supportive care needs in four domains: cancer information, daily living, behavioral health, and language assistance. Hierarchical cluster analysis was used to identify clusters of participants based on their supportive need profiles to further examine the association between need profiles and quality of life (QoL) assessed by the Functional Assessment of Cancer Therapy.

Participants (mean age = 57.6) included 72% males and 62% spoke English less than very well. Older participants (age ≥ 65) and those with annual income <$50K reported higher daily living needs. Men and younger participants (age < 50) reported higher behavioral health needs. We found three clusters displaying distinct cancer supportive need profiles: Cluster 1 (28% of the sample) displayed high needs across all domains; Cluster 2 (51%) had low needs across all domains; and Cluster 3 (21%) had high needs for cancer information and daily living. Cluster 1 participants reported the lowest QoL.

**Conclusion:**

Cancer supportive care needs among Asian American patients with colorectal, liver, and lung cancer were associated with patient characteristics and QoL. Understanding cancer supportive care needs will inform future interventions to improve care and QoL for Asian American patients with cancer. ClinicalTrials.gov Identifier: NCT03867916.

## 1 INTRODUCTION

Cancer is the leading cause of death for Asian Americans, with lung, colorectal, and liver cancers among the top five leading causes of cancer deaths for both men and women.^1^ Despite the high burden of cancer among Asian Americans,^2^ our understanding of cancer supportive care needs remains limited in this population,^3^, ^4^, ^5^, ^6^ which represents a critical gap in knowledge. Cancer supportive care is a person‐centered approach that connects patients with services to meet their informational, daily living, and emotional needs throughout their cancer trajectory, from diagnosis to survivorship.^7^, ^8^, ^9^ Addressing these needs can help improve patients' quality of life (QoL)^10^ and ultimately decrease cancer recurrence and mortality.^11^, ^12^, ^13^ Some demographic factors such as younger age, higher education levels, and an annual income below $75 000 have been associated with heightened unmet supportive care needs,^14^, ^15^ potentially leading to diminished QoL.^10^ Self‐reported QoL among patients with cancer has an established association with cancer recurrence and mortality,^11^, ^12^, ^13^ further underscoring its significance. Few studies have systematically examined needs among Asian American patients newly diagnosed with cancer. In order to improve cancer care among this population with a high cancer burden, it is essential to gain a deeper understanding of cancer supportive care needs and their association with QoL among Asian American patients with cancer.

The Patient Cancer OUtreach, Navigation, Technology and Support (Patient COUNTS) Study provided linguistically and culturally‐sensitive patient navigation for Asian American patients with lung, colorectal, or liver cancer undergoing treatment. We previously described supportive care needs from the first phase of Patient COUNTS, which was an in‐person pilot.^16^ Utilizing data collected from the second phase of the Patient COUNTS study, which implemented a web‐based patient portal and virtual patient navigators to direct patients to resources, we aimed to further understand supportive care needs among Asian American patients with colorectal, liver, or lung cancer who are starting or undergoing treatment for cancer by:

1. examining supportive care needs in four major domains (cancer information, daily living, behavioral health, and language) and participant factors correlated with these needs;
2. identifying profiles of supportive care needs; and
3. examining whether the identified needs profiles are associated with QoL.

## 2 METHODS

### 2.1 Study design

The study utilized baseline data collected from Patient COUNTS, a single‐arm prospective cohort pilot study designed to test the feasibility and acceptability of a web‐based patient navigation intervention for Asian American patients newly diagnosed with colorectal, liver, or lung cancer (ClinicalTrials.gov Identifier: NCT03867916).

The program assigned each participant to a language‐concordant patient navigator—English, Chinese, or Vietnamese. The navigators were non‐healthcare professionals with experience in health education and who received training through the Shanti Project, a community organization with 40+ years of experience in training navigators.^17^ Over 6 months, navigators engaged with participants via phone, email, text, or WeChat, a popular social media application among Chinese American users.^18^ All research procedures were approved by the Institutional Review Board (#18‐25820) at the University of California, San Francisco and the state of California Committee for the Protection of Human Subjects (#2019‐176). Informed consent was obtained from all study participants. All consent and study materials were available in English, Chinese, and Vietnamese.

### 2.2 Recruitment

From February 2020 to November 2021, we recruited newly diagnosed patients with colorectal, liver, or lung cancer, whose diagnosis dates ranged from October 2019 to August 2021. Participants were identified using an early case ascertainment (ECA) process from the Greater Bay Area Cancer Registry (GBACR). The GBACR is a population‐based cancer registry that covers nine counties in the San Francisco Bay Area of California and is part of the National Cancer Institute's Surveillance, Epidemiology, and End Results program and the statewide California Cancer Registry.^19^ Consent was obtained over the phone or online. For interested participants who deferred to family members, we obtained verbal consent from the participant to allow us to speak to their family member.

The inclusion criteria were as follows: self‐identified as Asian or Asian American; aged 21 years or older; spoke English, Cantonese, Mandarin, or Vietnamese; had stage I–IV colon, rectum, liver, or lung cancer; received healthcare in one of nine Greater Bay Area counties; were currently receiving or planning to receive treatment; had access to or were willing to create an email account; and were willing to stay in the study for 6 months. The exclusion criteria were any medical or psychological conditions precluding informed consent, receiving institutionalized care (e.g., assisted living, hospice, and incarceration), or if the patient already completed treatment.

### 2.3 Web‐based patient portal

The study team worked with UCSF School of Medicine Technology Services to develop a web portal system to provide navigation to Asian American patients with cancer in English, Chinese, and Vietnamese. The portal was built on Salesforce, a secure, HIPAA‐compliant cloud‐based platform. After the participant completed the needs assessment survey on the portal, the navigator used the responses to send relevant resources to patients on the portal and address any questions or concerns the patient had.

### 2.4 Survey administration

After enrollment, participants completed a baseline survey and needs assessment. During the intervention, participants were asked to complete follow‐up surveys at 3 and 6 months as well as a user experience survey at 7 months. Surveys were conducted on the patient portal online or by telephone, based on participant's preference. Participants received a $25 gift card upon completion of each survey.

### 2.5 Measures

Cancer needs were assessed by asking participants to indicate “yes/no” for each of 15 items corresponding to a specific need across four domains, which were based on Evans Webb and colleagues' model^20^:

1. Cancer information needs (six items): cancer diagnosis and staging; cancer treatment options; coping with side effects such as fatigue and nausea; healthcare access for cancer care; nutrition and physical activity recommendations for cancer recovery; and talking with friends and family about cancer diagnosis;
2. Daily living needs (five items): financial matters related to cancer care; transportation; legal concerns; housing; and food access;
3. Behavioral health needs (three items): seeking help for mental health, emotions, or anxiety; smoking cessation resources; and information or resources related to alcohol or other substance use;
4. Language assistance need (one item): whether participants needed assistance in medical interpretation and translation.

We calculated each needs domain score by taking the sum of participant survey responses that indicated “yes” for each item in each domain. Then, we divided the sum by the total number of items in the domain. For example, the daily living domain had five items, so the sum of “yes” responses for the five items in this domain was divided by 5.

We assessed QoL using the Functional Assessment of Cancer Therapy‐General (FACT‐G), a 27‐item measure that is well‐established in assessing QoL in patients with cancer.^21^ The four FACT‐G subscales were: physical (seven items), social (six out of the seven items were used), emotional (six items), and functional well‐being (seven items). Each item scored from 0 (not at all) to 4 (very much); negatively worded items were reverse scored so that higher overall subscale scores indicate higher QoL. Because of an error in one of the social well‐being subscale items “I feel close to my friends,” where the word “friends” was replaced by “family” across language versions and we excluded that item from score computations.

Thus, the FACT‐G total score was computed based on 26 items and the social well‐being subscale score was computed from 6 items; there was no deviation in the computation of other subscale scores. The Cronbach's alphas of the FACT‐G subscale scores of the study sample ranged from 0.81 to 0.91, indicating acceptable internal consistency across subscales.

Other variables included in this study analyses were sociodemographics, which included age, sex, Asian ethnic group (Chinese, Vietnamese, Filipinx, or “Other”), preferred language for study participation (Chinese, Vietnamese, or English), self‐rated spoken English proficiency (“Not at all,” “Not well,” “Well,” or “Very well”), education, employment, household income, and marital status. Cancer‐specific variables included cancer diagnosis, staging, and treatment status.

### 2.6 Data analysis

Our analyses included participants who provided data on the baseline and needs assessment survey (*n* = 47). Using SPSS v27 (IBM), descriptive statistics and Pearson correlation coefficients were used to examine the bivariate associations between patient characteristics and needs domain scores. In addition, we conducted bivariate analyses and multivariable generalized linear models (GLM) to identify correlates for each needs domain. Age, sex, ethnicity (Chinese vs. other Asian), preferred language for study participation (English vs. non‐English), English proficiency, and cancer stage (early stage I/II; late III/IV; unknown) were included as a priori covariates in all GLM analyses. Additional covariates were included in the final multivariable models when bivariate analyses attained a *p*‐value ≤.05. A binary logistic regression analysis was used for the language domain score.

To identify cancer supportive care needs profiles, we performed a hierarchical cluster analysis using Ward's linkage with squared Euclidean Distance. The four needs domains were used as clustering variables; each was range standardized (from 0 to 1). We used a dendrogram (Figure S1) as a graphical representation of the possible clusters that can be created by Ward's method.^22^ To ensure that each cluster size was not less than seven participants,^23^ we considered solutions ranging from 2 to 4 clusters, examined their cluster sizes, and resulting profiles. A 3‐cluster solution was selected because it yielded clusters with the greatest number of needs profiles with group sizes of no less than seven participants. We further validated our findings by conducting analyses with additional methods: (1) complete linkage (hierarchical) and (2) K‐means (non‐hierarchical). These two additional methods replicated the 3‐cluster solution findings from Ward's method (Figures S2 and S3). Using final clusters as independent variables, we conducted a multivariate analysis of variance (MANOVA) for the four FACT‐G subscale scores, and an ANOVA for the FACT‐G total score to examine association between needs profile clusters and QoL measured by FACT‐G scores. We used ANOVAs as follow‐up analyses for the MANOVA for each subscale score separately. LSD post‐hoc pairwise tests were used when *p*‐value were ≤.05 to determine the association between needs clusters and QoL as measured by FACT‐G subscale and overall scores.

## 3 RESULTS

**FIGURE 1:**
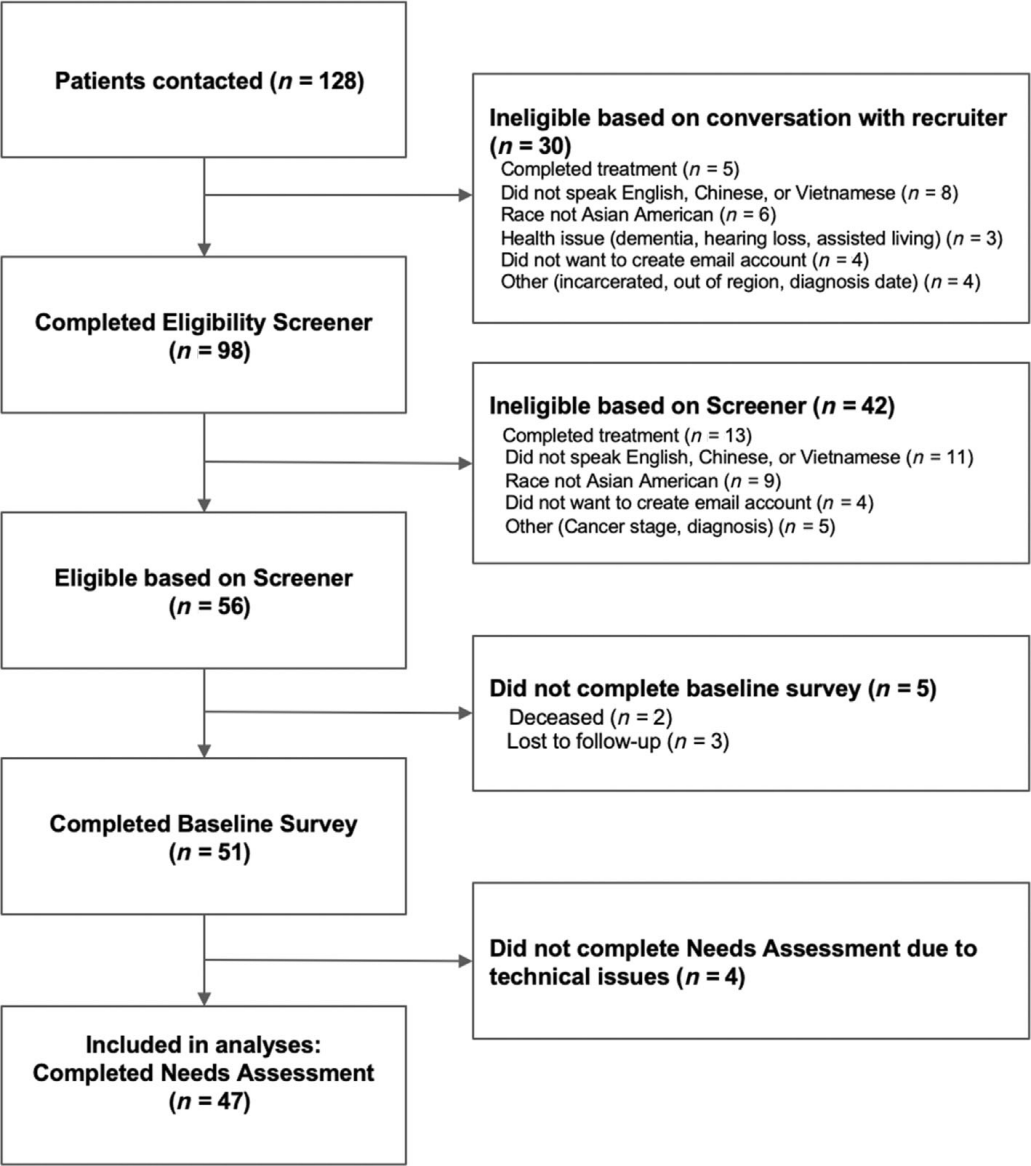
Participant flow diagram.

Figure 1 shows the participant flow diagram. The mean age was 57.6 (SD = 13.2; range: 30–82), 72% were men and 45% were Chinese (Table 1). In terms of preferred language for study participation, a majority (62%) of participants preferred participating in English, 32% preferred Chinese (Cantonese or Mandarin), and 6% preferred Vietnamese. A majority (62%) reported speaking English less than very well and 34% did not attend college. Participants had lung (43%), colon (53%), or liver (4%) cancer at stage I (19%), II (21%), III (26%), or IV (13%), including 21% with unknown cancer stage (Table 1).

**TABLE 1:** Characteristics of Asian American patients with colorectal, liver, and lung cancer who participated in Patient COUNTS (*N* = 47).

	Frequency	%
Sex		
Female	13	27.7%
Male	34	72.3%
Age^a^	Mean: 57.6	SD: 13.2
30–34	<5*	<10.6%
35–49	11	23.4%
50–64	15	31.9%
65–79	18	38.3%
80+	<5	<10.6%
Ethnicity^a^		
Chinese	21	44.7%
Other Asian^b^	26	55.3%
Preferred language for study participation		
Chinese (Cantonese or Mandarin)	15	31.9%
English	29	61.7%
Vietnamese	3	6.4%
English proficiency		
Very well	18	38.3%
Well	11	23.4%
Not well	10	21.3%
Not at all	8	17.0%
Education		
≤High school graduate or equivalent	10	21.3%
Some college or vocational training	6	12.8%
College graduate	13	27.7%
Graduate school (up to and including Masters degree)	11	23.4%
Graduate school beyond Masters (Doctorate degree)	5	10.6%
Prefer to not answer	2	4.3%
Employment		
Employed	20	42.6%
Not employed	11	23.4%
Retired	15	31.9%
Household annual income		
≤$20 000	8	17.0%
$20 001–$50 000	5	10.6%
$50 000–$100 000	11	23.4%
>$100 000	14	29.8%
Prefer to not answer or don't know	9	19.1%
Marital status		
Legally married or living together	39	83.0%
Separated, divorced, widowed, or single	5	10.6%
Other	1	2.1%
Prefer to not answer or don't know	2	4.3%
Cancer type^a^		
Colorectal	25	53.2%
Liver	<5	<10.6%
Lung	20	42.6%
Cancer stage		
I	9	19.1%
II	10	21.3%
III	12	25.5%
IV	6	12.8%
Don't know or missing	10	21.3%
Cancer treatment status (categories not mutually exclusive)		
Had surgery	32	68.1%
Started chemotherapy	26	55.3%
Started radiation therapy	15	31.9%
Did not start cancer treatment	2	4.3%

^a^ The ethnicity, age, and cancer type data were obtained from the Greater Bay Area Cancer Registry, which prohibits displaying individual data with <5 participants.

^b^ Other Asian ethnicities of participants included Filipino and Vietnamese American.

**FIGURE 2:**
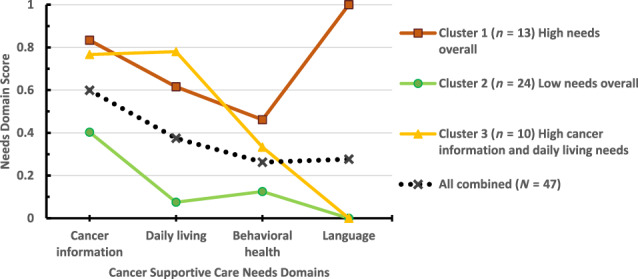
Cancer supportive care needs domain scores for all participants and by clusters.

The mean number of needs reported was 6.5 (SD = 4.1) out of 15. A majority (70%) reported needs in at least 2 domains. Cancer information was the most prominent needs domain (Figure 2).

Daily living needs were higher in those with annual income ≤$50 000 (*B* = 0.29; *p* = .03) compared to those with annual income >$50 000 and those who were 65+ years old (*B* = 0.32; *p* = .04) compared to those <50 years old (Table 2). However, daily living needs were lower among retirees (*B* = −0.55; *p* < .01) when compared to those who were unemployed. Men (*B* = 0.20; *p* = .04) and participants <50 years old (*B* = 0.24; *p* = .05) reported higher behavioral health needs. Cancer information needs were not associated with participant characteristics (Table 2).

**TABLE 2:** Correlates of cancer supportive care needs: results of multivariable generalized linear models (*N* = 47).

	Cancer supportive care needs domains											
	Cancer information			Daily living			Behavioral health			Language		
Participant characteristics	B	(95% CI)	*P*–value	B	95% CI	*P*–value	B	95% CI	*P*–value	B	95% CI	*P*–value
Age												
30 – 50	Referent			Referent			Referent			Referent		
50 – 64	0.05	(–0.19, 0.29)	0.66	–0.01	(–0.28, 0.25)	0.92	**–0.24**	**(–0.48, –0.003)**	**0.05**	–0.49	(–3.71, 2.73)	0.77
65 +	0.05	(–0.17, 0.28)	0.63	**0.32** ^a^	**(0.01, 0.64)**	**0.04**	–0.21	(–0.43, 0.02)	0.07	–1.01	(–4.3, 2.28)	0.55
Sex												
Female	Referent			Referent			Referent			Referent		
Male	–0.03	(–0.22, 0.16)	0.78	0.06	(–0.14, 0.26)	0.54	**0.20**	**(0.01, 0.39)**	**0.04**	–0.07	(–2.88, 2.73)	0.96
Ethnicity												
Chinese	Referent			Referent			Referent			Referent		
Other Asian	0.08	(–0.11, 0.26)	0.41	0.08	(–0.13, 0.28)	0.48	0.05	(–0.13, 0.24)	0.57	0.41	(–1.93, 2.74)	0.73
Cancer stage												
Stages 1 or 2	Referent			Referent			Referent			Referent		
Stages 3 or 4	–0.01	(–0.19, 0.18)	0.94	0.01	(–0.19, 0.20)	0.94	–0.06	(–0.25, 0.13)	0.54	–0.88	(–3.12, 1.36)	0.44
Unknown	0.03	(–0.19, 0.26)	0.78	0.03	(–0.21, 0.27)	0.80	0.17	(–0.06, 0.39)	0.15	0.83	(–3.67, 5.34)	0.72
English proficiency^b^	0.04	(–0.12, 0.04)	0.36	–0.01	(–0.11, 0.08)	0.79	–0.07	(–0.15, 0.01)	0.08	**–2.73**	**(–4.67, 0.79)**	**0.01**
Graduated from college		Not included			Not included			Not included				
No										Referent		
Yes										0.63	(–1.91, 3.18)	0.63
Employment status		Not included						Not included			Not included	
Unemployed				Referent								
Employed				–0.01	(–0.28, 0.25)	0.92						
Retired				**–0.55**	**(–0.88, –0.25)**	**<0.001**						
Annual household income		Not included						Not included			Not included	
> $50K				Referent								
< $50K				**0.29**	**(0.03, 0.55)**	**0.03**						
Unknown				0.04	(–0.24, 0.32)	0.77						

*Note*: Estimates attained *p* – value < 0.05 are denoted in bolded fonts.

^a^ Estimates and *p*–values < 0.05 are in bold.

^b^ English proficiency was modeled as a continuous variable from 0 (“not at all”) to 3 (“very well”).

A 3‐cluster solution was deemed optimal as guided by a dendrogram for grouping participants based on their needs: participants with high cancer supportive care needs across all domains (Cluster 1, *n* = 13, 27.7% of the sample); those with low needs across all domains (Cluster 2, *n* = 24, 51.1%); and those with high cancer information and daily living needs (Cluster 3, *n* = 10, 21.3%) (Figure 2). Cluster 1 participants reported higher scores on all the needs domains than Cluster 2 participants (*p* < .001). Cluster 1 and 3 participants were similar in all domains of needs (*p* > .05) except that all Cluster 1 participants indicated language assistance whereas no Cluster 3 participants indicated such needs. Cluster 2 participants also had lower needs scores than Cluster 3 participants for cancer information and daily living needs (*p* < .001) but were statistically similar on the needs for behavioral health and language (absence of language assistance). Cluster 3 participants were similar to the other two clusters in behavioral health needs (*p* > .05).

**FIGURE 3:**
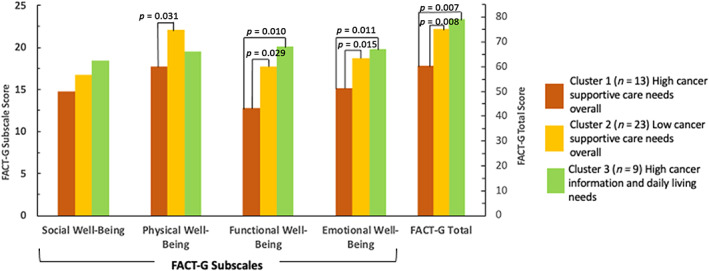
Functional Assessment of Cancer Therapy subscale and total scores for each cluster (*N* = 45). Analyses were based on a total of 45 participants who provided data on at least half of the FACT‐G items for subscales and total score computations. The *p*‐values denote pairwise comparisons between clusters using post hoc LSD tests that attained *p*‐values <.05.

Cluster 1 participants who showed high needs across all domains reported the lowest QoL compared with Clusters 2 (*p* = .008) and 3 participants (*p* = .007). Clusters 2 and 3 participants were similar in their total FACT‐G scores. Cluster memberships differed by the FACT‐G subscales (Roy's Largest Root. 0.273, *F* = 2.731, *p* = .04, partial *η* ^2^ = 0.215). Cluster 1 participants reported the lowest scores for all the FACT‐G subscales in general as well as reported lower scores than Cluster 2 participants for the physical, functional, and emotional well‐being subscales (*p* < .05) (Figure 3). Cluster 1 participants, when compared to Cluster 3 participants, also had lower scores on functional and emotional well‐being subscales (*p* < .05). Clusters 2 and 3 were similar on FACT‐G subscale scores. These three clusters were similar for social well‐being.

## 4 DISCUSSION

In our study of Asian American patients with cancer who were starting or undergoing treatment, we found that lower income, male sex, younger age, lower English proficiency, and being unemployed were associated with higher needs in daily living, behavioral health, or language assistance. We also identified three clusters of distinct needs profiles and these profiles were associated with unique QoL experiences. To our knowledge, this is the first study to identify patient factors that are associated with supportive cancer care needs and QoL among Asian American patients with cancer.

Previous studies investigating cancer supportive care needs utilized standardized questionnaires, such as the Supportive Care Needs Survey (SCNS),^24^ Comprehensive Needs Assessment Tool (CNAT),^25^ Cancer Needs Questionnaire (CNQ),^26^ and Needs Evaluation Questionnaire (NEQ).^27^ However, these standardized questionnaires do not include the need for language assistance, which is important to our sample of Asian American patients with cancer, many of whom indicated that they did not speak English well. Additionally, prior studies on supportive care needs among patients with cancer did not include an assessment of language assistance needs.^28^, ^29^, ^30^ For our study, we modified the model of supportive care needs domain by Evans Webb and colleagues to create the needs domains which are inclusive of the language assistance need of the Asian American patients in our sample.^20^

In our study, daily living needs were higher for those with lower income, and behavioral health needs were higher among male and younger participants. Our findings are similar to prior studies that reported higher cancer‐related distress in young adults compared to senior adults but similar distress levels to middle‐aged adults.^15^ Our results are consistent with another study that found that younger (<55 years old), unmarried, and low income women with early‐stage breast cancer after surgery had higher psychological risk.^14^ However, these previous studies included very few Asian American patients. Our study findings support that daily living needs should be especially assessed and addressed for Asian American patients who have lower income, are younger than 50 years old, and are male.

Our findings highlight the importance of providing Asian American patients with comprehensible information relevant to their cancer care on diagnosis and staging, treatment options, and nutrition and physical activity recommendations for cancer recovery. In addition to utilizing patient navigators, interventions utilizing online and web‐based tools to deliver information and resources to patients, especially those with limited English proficiency (LEP), could be efficient and effective. The pandemic led to an expansion in telehealth use in medicine due to its ease of access.^31^ There is a need for more studies to investigate the feasibility of web‐based tools among Asian Americans who have LEP.

Our cluster analysis revealed that there were three distinct groups among our participant sample based on their cancer supportive care needs. Each group had a distinct profile of needs across the cancer supportive care needs domains: high needs across all domains (Cluster 1), low needs across all domains (Cluster 2), and high needs for cancer information and daily living (Cluster 3). The cluster that reported high needs across all domains (Cluster 1) included participants with the most language assistance needs, while the other clusters (Clusters 2 and 3) included exclusively participants who did not indicate the need for language assistance. Asian American patients in our sample who indicated language needs also had high needs in other domains. In a 2017–2019 national survey,^32^ almost one in three (28%) Asian Americans reported being “less than proficient” in English and two‐thirds spoke a language other than English at home.^32^, ^33^, ^34^ Not speaking English well^35^, ^36^ is associated with poor health outcomes due to challenges in navigating the healthcare system in the United States.^1^, ^37^, ^38^ Asian Americans with LEP are two times more likely not to have a primary care provider and almost five times more likely to have communication challenges in healthcare settings compared to Asian Americans who are proficient in English.^33^ Language barriers could further exacerbate a patients' needs in other areas of cancer supportive care as shown by the clusters identified in the study. These findings underscore the importance for cancer care interventions to increase the accessibility of language translators and multilingual patient navigators in clinical settings as well as translated health informational materials in order to better serve Asian American patients with cancer with LEP.

In addition, distinct cancer supportive needs profiles were associated with differences in QoL. We anticipated that patients with more supportive care needs would have lower QoL because of unmet needs in their cancer treatment and recovery process. Specifically, the cluster that reported high needs in all domains (Cluster 1) also reported lower QoL (physical, functional, emotional, and overall well‐being). However, the cluster reporting lower needs overall (Cluster 2) had a similar QoL (social, physical, functional, emotional, and overall well‐being) as the cluster with high cancer information and daily living needs (Cluster 3). Additionally, patients across the three clusters had similar social well‐being QoL regardless of having low or high supportive care needs. One explanation for these results is that the process of navigating cancer treatment and recovery can come with the experiences of social isolation (decreased social well‐being),^39^ adverse treatment side effects/symptoms (decreased physical well‐being),^40^, ^41^ disruption in daily work and routines (decreased functional well‐being),^42^, ^43^ and psychological distress (decreased emotional well‐being)^44^ for many patients regardless of their baseline supportive care needs. The cancer supportive care need profiles identified in this study should be considered as preliminary given the exploratory nature of cluster analytic approaches and the small sample size. Nonetheless, the innovative use of cluster analyses provided initial evidence of the associations between patterns of unmet cancer supportive care needs and QoL across social, physical, functional, emotional, and overall well‐being. These findings help inform future research that investigates how Asian American patients' varying level of cancer supportive care needs influences their QoL and clinical outcomes.

Our study has several limitations. First, our sample size was small (*N* = 47). Although our sample of Asian American participants included multiple Asian ethnic groups, varying levels of English proficiency, and had various profiles of supportive care needs, our findings remain exploratory and cannot be generalized to all Asian American patients receiving cancer treatment. Similarly, findings cannot be applied to younger patients with cancer given that more than 80% of the study sample was older than 50 years of age. The need for language assistance was assessed by a single item; multiple items would have allowed for a more comprehensive assessment including specific types of language assistance such as medical interpretation, translation, and both verbal and written communication needs. Similarly, our assessment of language proficiency only focused on spoken English and did not include other aspects of proficiency in reading or writing. An individual proficient in spoken English might still need assistance in reading and comprehending written instructions from their healthcare providers, and we did not assess this. Given our results, future research should investigate different modalities of language assistance. Because our study used ECA and updated clinical staging from the registry was not available at the time of our analysis, we had a larger than anticipated percentage of missing cancer stages. Therefore, our study had limited power to determine the associations between cancer stages and cancer supportive needs, but our analyses nonetheless identified significant correlates of cancer supportive needs beyond cancer stage. Lastly, the FACT‐G total and its Social/Family Well‐Being subscale scores reported in this study excluded an item “GS1 ‘I feel close to my friends’” due to a technical error, which might make this subscale and overall FACT‐G score not comparable to other studies using FACT‐G scales.

## 5 CONCLUSION

Asian American patients with cancer in this study reported supportive care needs in multiple domains, with cancer information being most prominent. Patients' needs of cancer supportive care were associated with patient characteristics as well as QoL, which underscore the importance of providing a patient‐centered approach to individualize navigation of resources to meet Asian American patients' cancer supportive care needs in multiple areas. These findings will inform future interventions to improve care and QoL for Asian American patients with cancer.

## AUTHOR CONTRIBUTIONS

**Katarina Wang:** Data curation (equal); formal analysis (equal); validation (equal); writing – original draft (lead); writing – review and editing (equal). **Janet Chu:** Data curation (equal); formal analysis (equal); supervision (supporting); validation (equal); writing – review and editing (equal). **Debora L. Oh:** Investigation (equal); supervision (equal); validation (equal). **Salma Shariff‐Marco:** Conceptualization (equal); funding acquisition (equal); methodology (equal); project administration (equal); supervision (equal); writing – review and editing (equal). **Laura Allen:** Investigation (equal); project administration (equal); resources (equal); supervision (equal); validation (equal); writing – review and editing (equal). **Mei‐Chin Kuo:** Investigation (equal); project administration (equal); resources (equal); supervision (equal); validation (equal); writing – review and editing (equal). **Ching Wong:** Investigation (equal); project administration (equal); supervision (equal); writing – review and editing (equal). **Hoan Bui:** Investigation (equal); resources (equal); writing – review and editing (equal). **Junlin Chen:** Investigation (equal); supervision (equal); writing – review and editing (equal). **Feng Ming Li:** Writing – review and editing (equal). **Carmen Ma:** Writing – review and editing (equal). **Angeline Truong:** Writing – review and editing (equal). **Scarlett L. Gomez:** Funding acquisition (equal); methodology (equal); project administration (equal); supervision (equal); writing – review and editing (equal). **Tung Nguyen:** Conceptualization (equal); data curation (supporting); funding acquisition (lead); investigation (equal); resources (equal); supervision (supporting); writing – review and editing (equal). **Janice Y. Tsoh:** Conceptualization (lead); data curation (supporting); formal analysis (equal); funding acquisition (supporting); investigation (equal); methodology (lead); software (equal); supervision (lead); validation (equal); visualization (equal); writing – original draft (supporting); writing – review and editing (lead).

## CONFLICT OF INTEREST STATEMENT

The team received funding from the Bristol Myers Squibb Foundation to conduct the project.

## ETHICS STATEMENT

This study approved by the Institutional Review Board at the University of California, San Francisco and the state of California Committee for the Protection of Human Subjects.

## Supporting information


**Figure S1:** Dendrogram using (A) Ward's Method; (B) Complete Linkage.
**Figure S2:** Cancer supportive care needs domain scores by clusters (A) Ward's Method Hierarchical Clustering; (B) Complete Linkage Hierarchical Clustering; and (C) K‐Means.
**Figure S3:** FACT‐G subscale and total scores for each cluster (A) Ward's Method Hiearchical Clustering; (B) Complete Linkage Hierarchical Clustering; and (C) K‐Means.

## Data Availability

The data that support the findings of this study are available on request from the corresponding author. The data are not publicly available due to privacy or ethical restrictions.
