# Supplementary material for: Correlates of supportive care needs among Asian Americans with colorectal, liver, or lung cancer from a web‐based patient navigation portal intervention: The Patient COUNTS study
Source: Cancer Rep (Hoboken). 2024 Feb 13;7(2):e1971. doi: 10.1002/cnr2.1971 (PMC10864727; doi:10.1002/cnr2.1971)

Figure S1: Dendrogram using A) Ward's Method B) Complete Linkage

A)

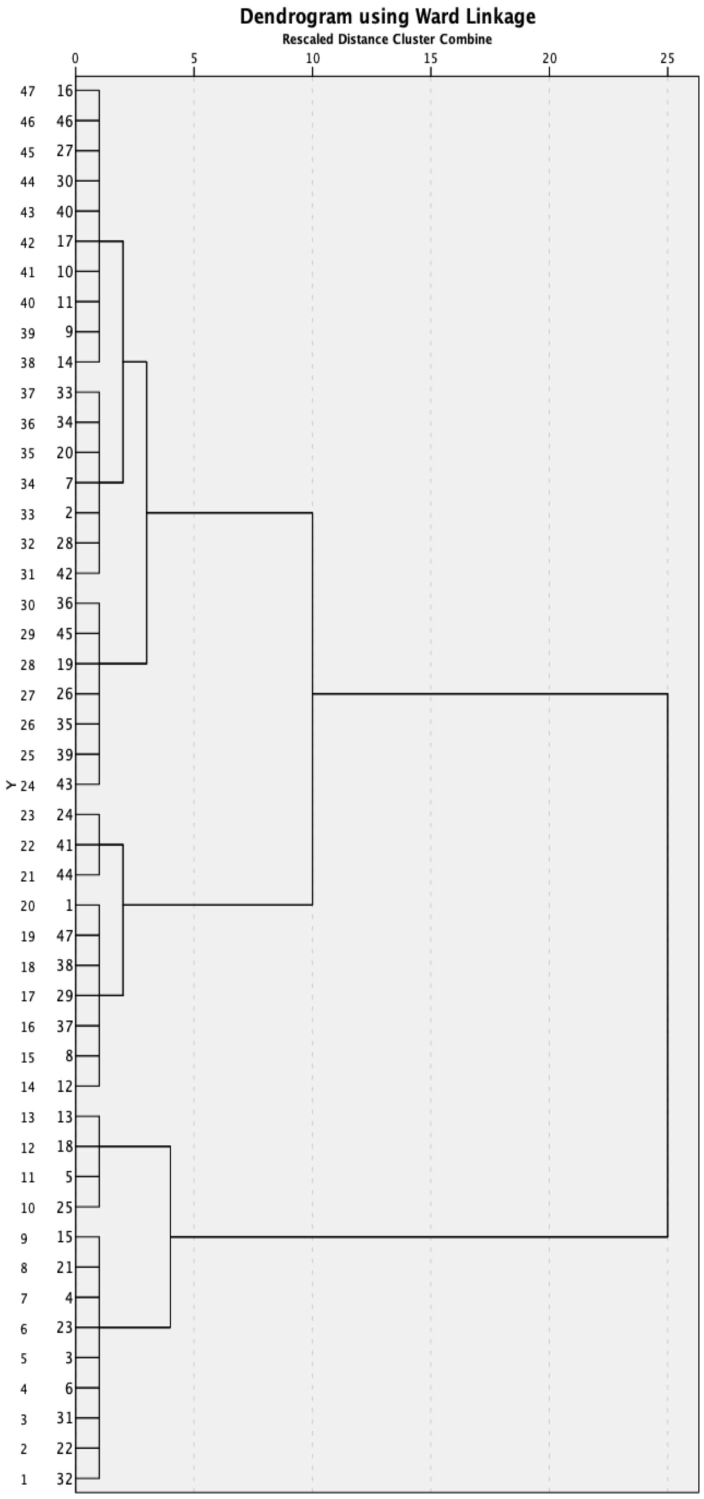

B)

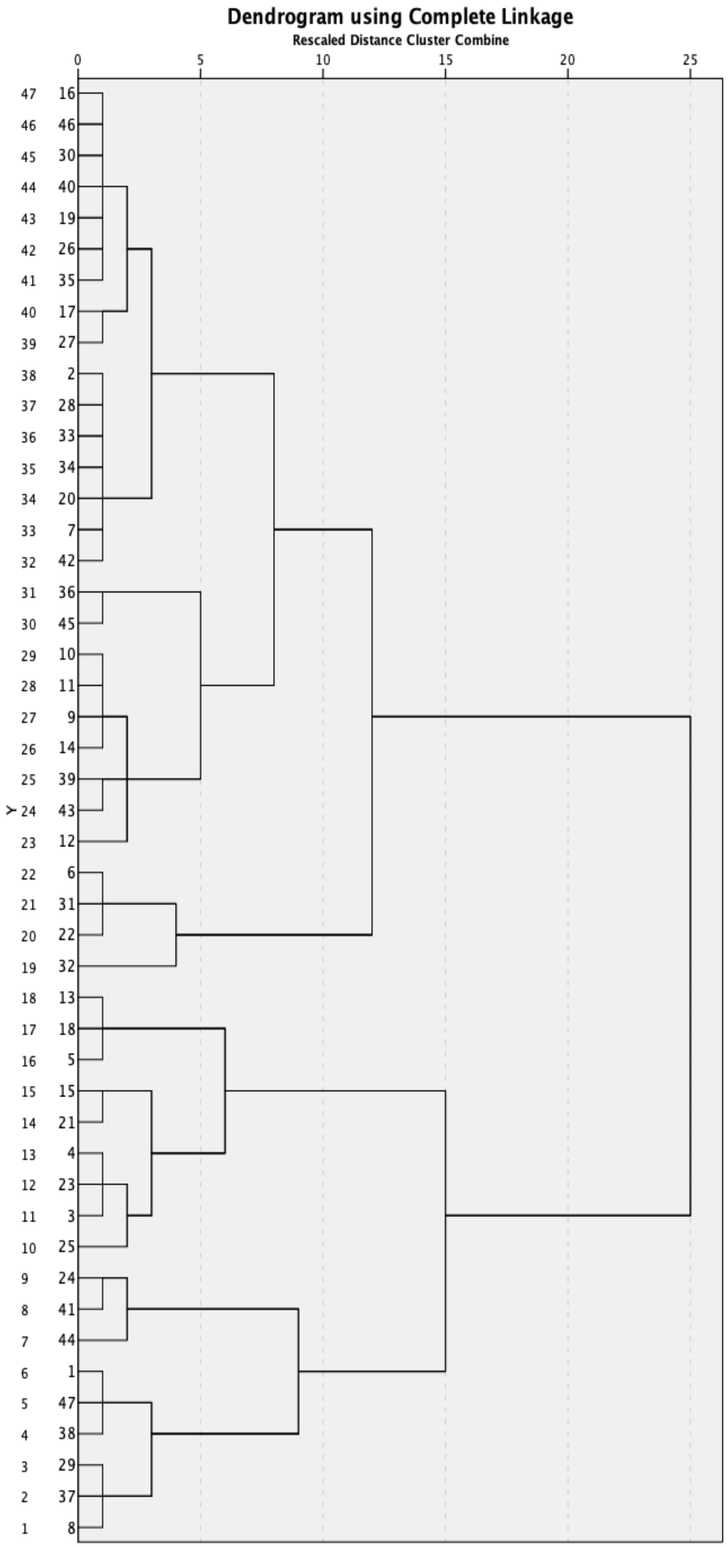

Dendrogram using Complete Linkage
Rescaled Distance Cluster Combine
0
5
10
15
20
25
Y

Figure S2: Cancer supportive care needs domain scores by clusters A) Ward's Method Hierarchical Clustering; B) Complete Linkage Hierarchical Clustering; and C) K-Means

A)

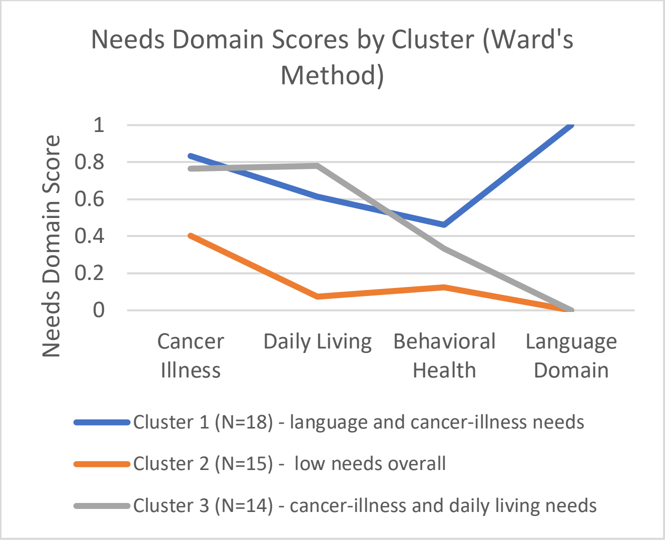

B)

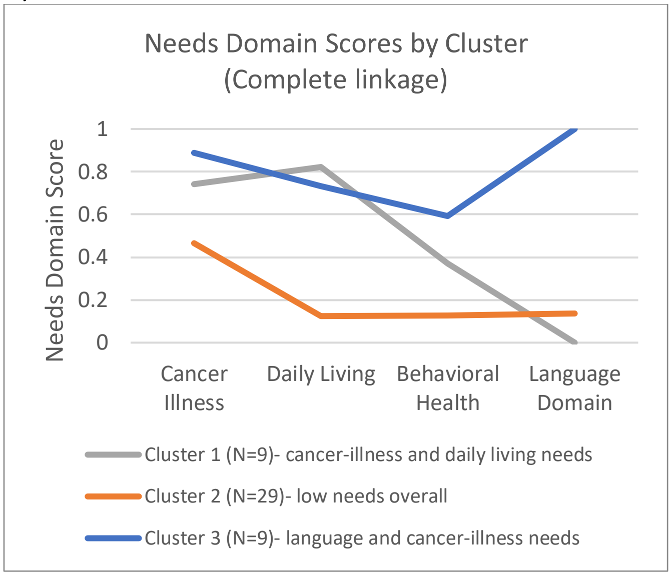

C)

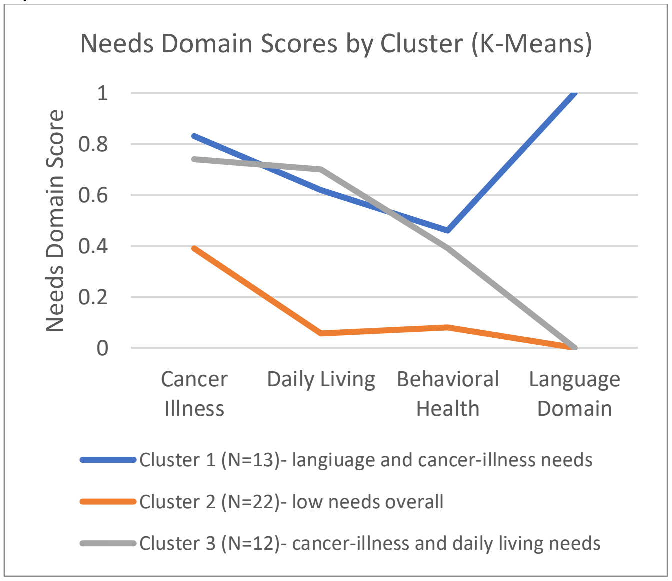

Figure S3: FACT_G subscale and total scores for each cluster A) Ward's Method Hierarchical Clustering; B) Complete Linkage Hierarchical Clustering; and C) K-Means

A)

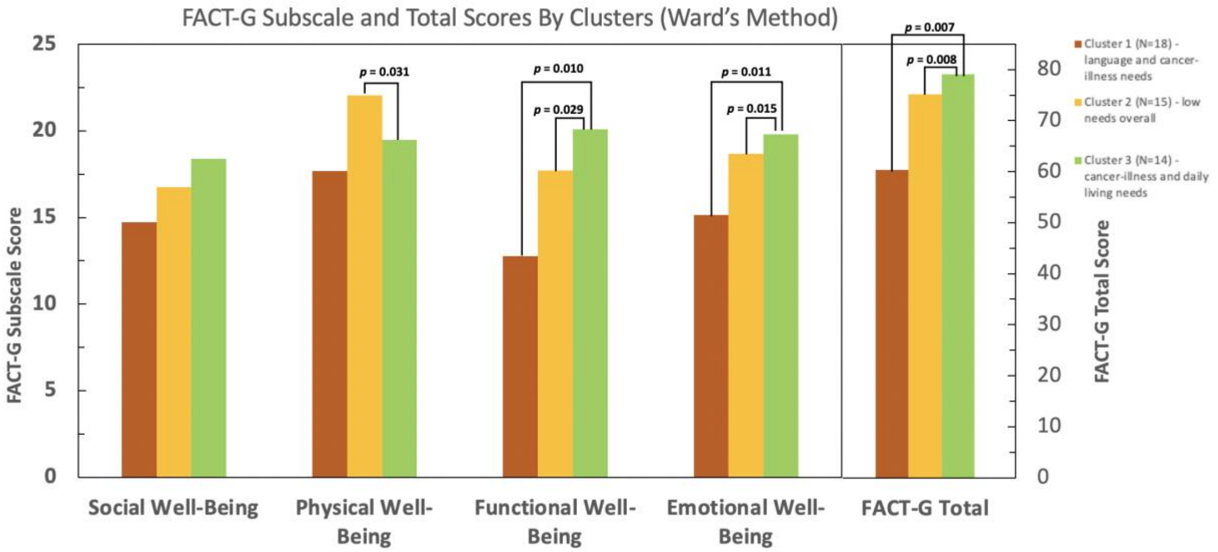

B)

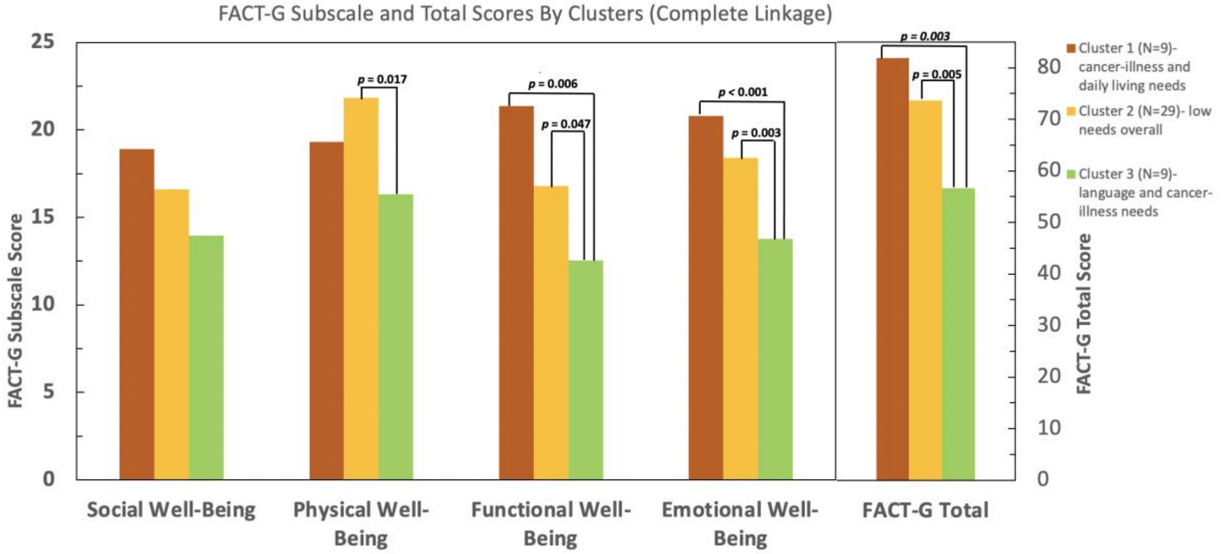

C)

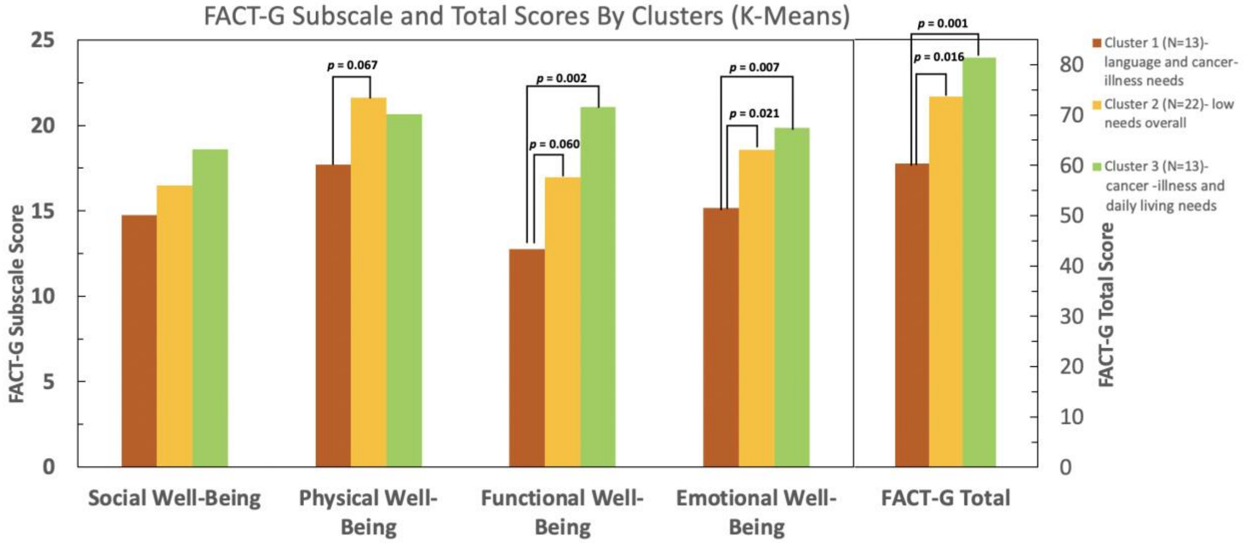

Supplement: Supplementary file 1 — Figure S1: Dendrogram using (A) Ward's Method; (B) Complete Linkage. Figure S2: Cancer supportive care needs domain scores by clusters (A) Ward's Method Hierarchical Clustering; (B) Complete Linkage Hierarchical Clustering; and (C) K‐Means. Figure S3: FACT‐G subscale and total scores for each cluster (A) Ward's Method Hiearchical Clustering; (B) Complete Linkage Hierarchical Clustering; and (C) K‐Means. [file CNR2-7-e1971-s001.pdf]
